# Identifying Tumor Cell Growth Inhibitors by Combinatorial Chemistry and Zebrafish Assays

**DOI:** 10.1371/journal.pone.0004361

**Published:** 2009-02-05

**Authors:** Jing Xiang, Hongbo Yang, Chao Che, Haixia Zou, Hanshuo Yang, Yuquan Wei, Junmin Quan, Hui Zhang, Zhen Yang, Shuo Lin

**Affiliations:** 1 Key Laboratory of Bioorganic Chemistry and Molecular Engineering of Ministry of Education and Beijing National Laboratory for Molecular Science (BNLMS), College of Chemistry, Peking University, Beijing, China; 2 Laboratory of Chemical Genomics, Shenzhen Graduate School, Peking University, Shenzhen, China; 3 Center of Developmental Biology and Genetics, College of Life Sciences, Peking University, Ministry of Education, Beijing, China; 4 State Key Laboratory of Biotherapy and Cancer Center, West China Hospital, West China Medical School, Sichuan University, Chengdu, China; 5 Nevada Cancer Institute, Las Vegas, Nevada, United States of America; 6 Department of Molecular, Cell, and Developmental Biology, University of California Los Angeles, Los Angeles, California, United States of America; Center for Genomic Regulation, Spain

## Abstract

Cyclin-dependent kinases (CDKs) play important roles in regulating cell cycle progression, and altered cell cycles resulting from over-expression or abnormal activation of CDKs observed in many human cancers. As a result, CDKs have become extensive studied targets for developing chemical inhibitors for cancer therapies; however, protein kinases share a highly conserved ATP binding pocket at which most chemical inhibitors bind, therefore, a major challenge in developing kinase inhibitors is achieving target selectivity. To identify cell growth inhibitors with potential applications in cancer therapy, we used an integrated approach that combines one-pot chemical synthesis in a combinatorial manner to generate diversified small molecules with new chemical scaffolds coupled with growth inhibition assay using developing zebrafish embryos. We report the successful identification of a novel lead compound that displays selective inhibitory effects on CDK2 activity, cancer cell proliferation, and tumor progression in vivo. Our approaches should have general applications in developing cell proliferation inhibitors using an efficient combinatorial chemical genetic method and integrated biological assays. The novel cell growth inhibitor we identified should have potential as a cancer therapeutic agent.

## Introduction

Cancer cell proliferation resembles normal embryonic growth in a way that both are extremely rapid. In zebrafish, a single cell zygote develops into an organism possessing essentially all organ rudiments of a vertebrate species in 24 hours. To achieve rapid cell growth, both developing embryonic cells and cancel cells use a strategy in which G1 and G2 phases of cell cycles are shortened or eliminated. Cyclin-dependent kinases (CDKs) play key roles in regulating cell cycle progression and their abnormal activation frequently associates with human cancers. CDKs are serine/threonine kinases that activate host proteins through phosphorylation on serine or threonine using adenosine triphosphate (ATP) as a phosphate donor. The activity of each CDK depends on the binding of a cognate cyclin[1], [2]. Although CDKs are continuously expressed, the concentration of cyclins are regulated by the cell cycle-dependent synthesis and ubiquitin-mediated degradation during the cell cycle[3]–[5]. The oscillation of CDK activities regulates cell cycle progression in response to a wide array of cell signaling pathways.

Altered cell cycles resulting from abnormal levels or activation of cyclins and CDKs occur frequently in human cancers[6]. Over-expression of cyclin E is observed in many human cancers including breast, brain, endometrial, and lung cancers, as well as lymphomas and leukemias[7]–[9]. The cyclin D1 gene is amplified in 15% of breast cancers and up-regulation of cyclin D1 is associated with large fractions of breast, ovarian, and other cancers[10], [11]. Abnormal activation of cyclin A is found in human hepatocarcinomas[12]. CDK2 normally associates with cyclin E or cyclin A and serves as a key regulator for the G1 and S phase progression[6] while CDK4 or CDK6 regulates G1 progression by interacting with cyclin D. The CDK2-cyclin E complex primarily regulates the G1 to S phase transition[13]–[15] whereas CDK2-cyclin A promotes S phase progression and drives its completion[16]. As CDKs are critically involved in regulating the cell cycle and their abnormal activities contribute to tumor genesis, often through interaction with pathways regulated by oncogenes and tumor suppressors, they have become valid targets for developing chemical inhibitors for cancer therapies[17]–[19].

To date, several small molecules that inhibit CDK2 activities have been identified[20]–[23]. Most of them induce cell cycle arrest at G1 phase, leading to either the inhibition of cell proliferation or induction of apoptosis in tumor cells. Several reports also showed that cells could be arrested at G2/M phases when treated with CDK2 inhibitors. Most encouragingly, some of these agents have been shown to induce tumor regression *in vivo* without significant toxicity to normal organisms[24]. Despite these findings, it is generally accepted that combinatory usage of inhibitors against various CDKs may be needed to fully block cancer proliferation since potential redundancy of CDK functions in the cell cycle may limit the effects of selective CDK inhibition. Therefore, it is highly desirable to expand the repertoires of new methods and screening strategies for rapidly synthesizing combinatorial chemicals and efficiently identifying active small molecular inhibitors for various CDKs.

Protein kinases share a highly conserved ATP binding pocket at which the majority of chemical inhibitors bind. Therefore, a major challenge in developing kinase inhibitors is achieving target selectivity. A critical factor towards selectivity is the development of synthetic methods that allow for the creation of focused chemical libraries with greater structure diversity. Diversity is an important parameter because it enables the identification of selective inhibitors across a panel of different kinases and simultaneously provides structure-activity information. By further improving chemical structures coupled with activity assays, this should facilitate the discovery and development of potent yet selective inhibitors for a desired class of protein kinases.

In connection with our development of a chemical genetic approach to analyzing biological systems by using interfacing libraries of small molecules followed by validating biological assays[25], we developed a highly efficient one-pot-synthesis[26]–[28] *via* a multi-components reaction[29]–[32] to generate focused chemical libraries. More importantly, we coupled the chemical approach to the whole zebrafish embryonic assay to rapidly select active molecules that inhibit growth and induce cell cycle arrest. Zebrafish embryos are externally accessible and their developmental growth is extremely rapid, reminiscent of tumor progression except in a highly controlled fashion. The simple developmental retardation assay of embryonic growth followed by determining stage of cell cycle arrest and apoptosis makes it possible to quickly identify inhibitors specific to cell cycle phases. Additionally, this system allows selection of less toxic compounds that do not cause necrosis of whole embryonic body. Further studies using chemical bioinformatics and biochemical assays suggested that the lead compound selected by zebrafish assay had a higher specificity to CDK2 kinase inhibition and it also reduced tumor cell proliferation *in vivo* without significant toxicity to xenograft mouse hosts.

## Results

### Computational design of CDK inhibitors

**Figure 1:**
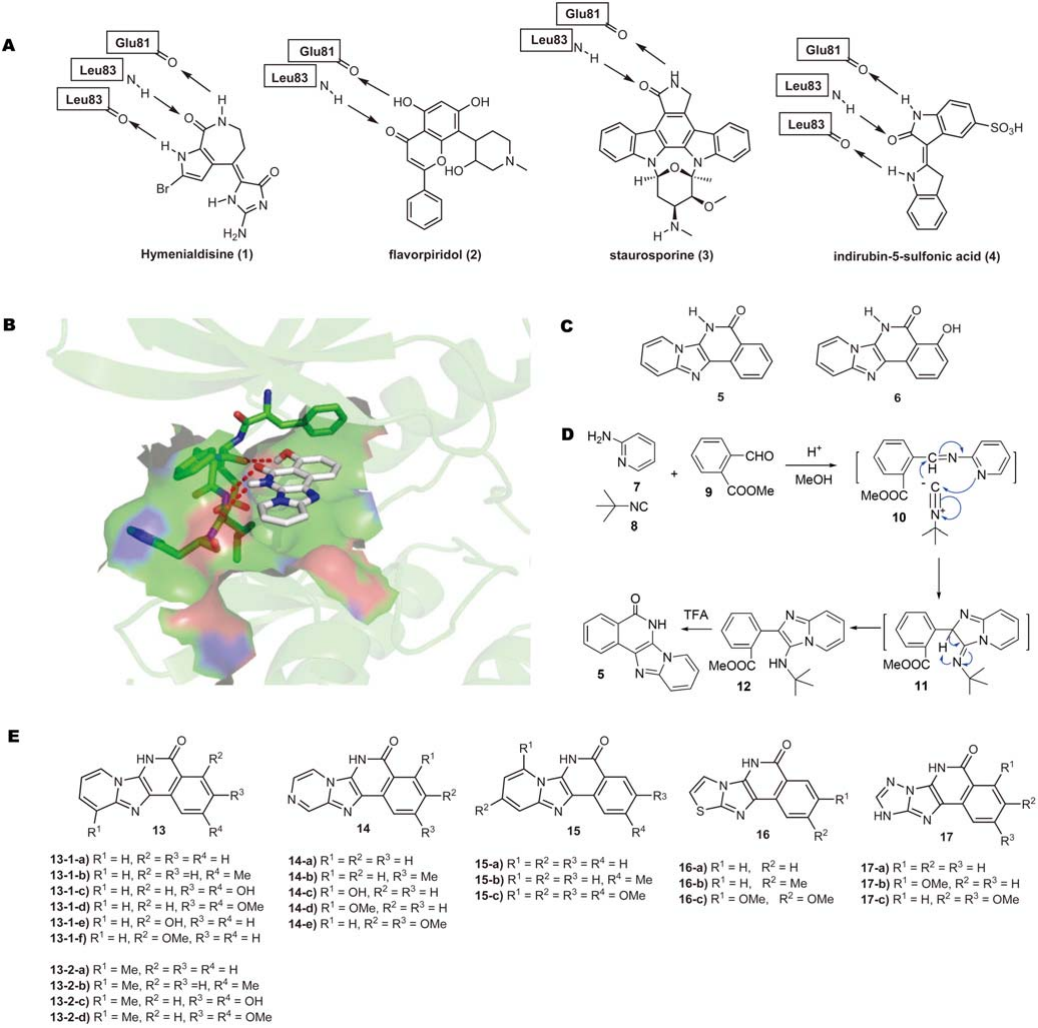
Structures, binding models and syntheses of CDKs inhibitors. (A) CDKs inhibitors and their bonding modes. (B) Docking of scaffold 6 with the crystal structure of CDK2 (PDB code: 1OI9) (docking software: AutoDock3.0). (C) Representative scaffolds (5 and 6) in docking. (D) Proposed one-pot synthesis of compound 5. (E) Synthesized quinoline-based polyheterocycles.

To date, most kinase inhibitors target the ATP-binding site. However, the ATP-binding pockets of 518 human kinases discovered so far are very similar to each other, especially for those kinases of the same superfamily or subfamily such as CDKs. The identification and synthesis of selective small-molecule kinase inhibitors was therefore regarded as a challenge and has been an active topic. Several kinase inhibitors have been identified, including staurosporine (3) and indirubin-5-sulfonic acid (4) (Figure 1A). These inhibitors can inhibit various CDKs by targeting the ATP binding pocket of CDKs, which is located in the deep cleft formed by N-lobe, C-lobe, and the hinge region in CDKs[1], [33]. Despite striking chemical diversity [34]–[36], those CDK inhibitors share several common features: (1) they act by competing with ATP for binding in the ATP-binding site; (2) they are flat, hydrophobic heterocycles; and (3) they bind mostly by hydrophobic interactions and hydrogen bonds with kinases. As a result, the cross-reactivity of these kinase inhibitors to a spectrum of other kinases prohibits their utilities as specific CDK inhibitors for cancer therapy.

To develop more specific CDK inhibitors, we focused our computational design on the common structural properties of these kinase inhibitors and the structural features of the ATP binding pocket of CDKs. Almost all of the CDK inhibitors form hydrogen bonds with the hinge region of CDKs, so we set this as the primary criteria to evaluate many known and our virtually designed scaffolds on the crystal structure of CDK2[37] (PDB code: 1OI9) using docking software, AutoDock3.0 (Figure 1B)[38]. Our examination revealed that a novel scaffold (5) in Figure 1C might potentially bind to CDK2 with high affinity (Estimated binding free energy ΔG∼8.6 kcal/mol). This scaffold satisfies the hydrogen bond criteria, and also has other common structural features of reported CDK inhibitors, like a planar hydrophobic heterocyclic framework, which fits well with the ATP binding cleft through favorable van der Waals and hydrophobic contacts. This scaffold has not been previously used for CDK2 inhibition and may provide a new scaffold for CDK inhibition. These quinoline-based poly-heterocycle scaffolds were further diversified and examined for potential high affinity and selectivity for CDK2. One of them, scaffold 6 (Figure 1C), can be designed with the intention of providing an additional phenolic group at the D ring to add the third hydrogen bond with the carbonyl group of Glu81. The binding model of this particular scaffold is similar to that of Flavopiridol (Figure 1A), an experimental drug currently in clinic trials, with an additional hydrogen bond between the N-H group of the lactam and carbonyl group of Leu83. Thus, the relatively small and novel structures of the quinoline-based poly-heterocycles provide a wide array of structural diversity for developing new specific CDK inhibitors. With these considerations, we synthesized a series of chemical compounds.

### Synthesis

To date, many heterocyclic scaffolds have been developed as kinase inhibitors[39]–[42], and each scaffold presents unique opportunities for the presentation of functional groups to the kinase active site. However, synthesis of those compounds usually requires lengthy synthetic routes with overall low yields, which prevents the syntheses of their structurally diverse analogs efficiently, and limits the feasibility to achieve the molecular libraries with discriminative binding to CDKs. To profile the kinase inhibitors covering the entire human kinome, there is a need to develop efficient and flexible methods for preparing novel and structural diverse of molecular libraries.

For the synthesis of our proposed novel scaffold 5, we intended to apply bienaymé's three-component reaction[43] of 2-aminopyrimidine 7, isonitrile 8, and aldehyde 9 to generate compound 12 through intermediates 10 and 11 (Figure 1D), which without purification could undergo the TFA-mediated intramolecular amide formation to afford quinoline-based tetracycle core, realizing a post-transformation strategy to quickly access 5 *via* a one-pot procedure[26]–[28].

Based on this highly efficient route, six different classes (24 compounds) of quinoline-based tetracycles were made from the commercially available or synthetically accessible materials (Figure 1E)[44]. Among them, scaffold 13-1 was predicted to have the highest affinity to CDK2 ATP-binding pocket; compounds bearing scaffold 13-2 to 17 were also synthesized to test our prediction model and to deduce the structure-activity relationship.

### Identifying growth inhibitors with selectivity to CDK2

Zebrafish embryos develop extremely rapidly and are highly accessible for direct microscopic observation. Several previous studies have taken such advantages of zebrafish and established that their embryos are useful as a whole animal screen model for specific chemical compound activities[45], [46]. Using zebrafish embryos one can visually examine desirable activity as well as toxicity of a compound. During early development, cell proliferation is very active and cells can complete the entire cycles in ranges of minutes[47]. We reasoned that if a compound can inhibit rapid growth of a zebrafish embryo, it would likely inhibit other rapid cell growth such as uncontrolled proliferation of cancer cells. In addition, since we can easily obtain primary cells from live embryos it is relatively straightforward to determine at which particular stage the cells are arrested, such as G1 or G2 phase.

**Figure 2:**
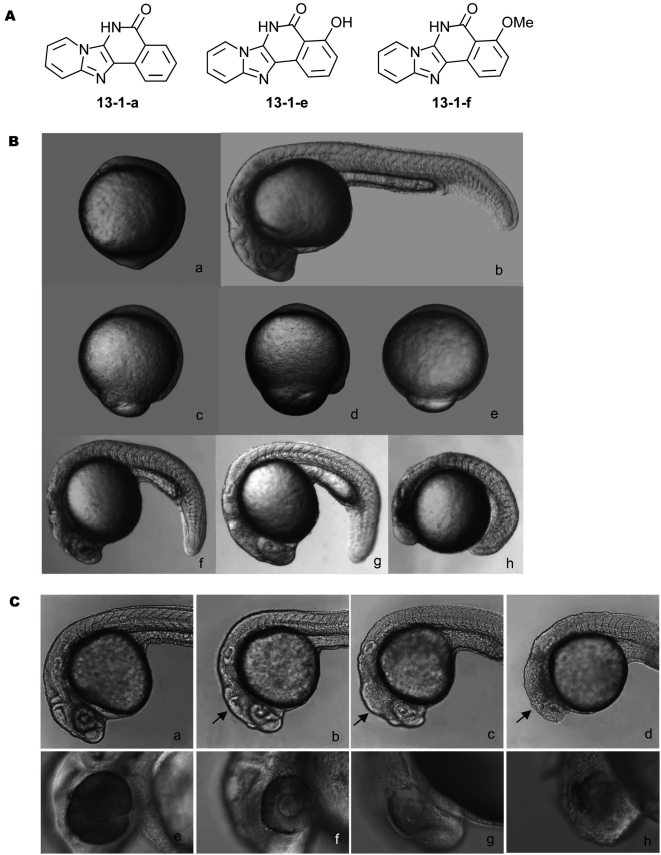
Representative active compounds and resulted phenotypes. (A) Representative active compounds. Compounds 13-1-a, 13-1-e and 13-1-f are the most potent in zebrafish assay with the lowest effective concentration below 10 µM. (B) Embryonic phenotypes of compound treatment. a) control (10 hpf), b) control (24 hpf); c–e) show embryos treated with 20 µmol/L of 13-1-a (c), 13-1-e (d), and 13-1-f (e) and observed at 10 hpf (compounds were added at 5.5 hpf); f–h) show embryos at 24 hpf after the same treatment [13-1-a (f), 13-1-e (g), 13-1-f (h)]. Note smaller sizes compared control. (C) Compound 13-1-e has stronger effect on brain and eye development of zebrafish embryos. a) control (24 hpf), b) 13-1-e (20 µmol/L), c) 13-1-e (30 µmol/L), d) 13-1-e (40 µmol/L), 24 hpf; e) control (36 hpf), f) 13-1-e (20 µmol/L), g) 13-1-e (30 µmol/L), h) 13-1-e (40 µmol/L), 36 hpf. Compounds were added at 5.5 hpf.

The 24 compounds described above were screened against zebrafish embryos and several compounds were shown to delay embryonic development without causing drastic change of body structure at concentrations of micromoles (Figure 2B). Three compounds (13-1-a, 13-1-e and 13-1-f) were selected for further studies due to their high potency (Figure 2A). To validate the zebrafish assay, we performed an independent cell proliferation assay by treating breast cancer cell line MDA-MB-231 with various concentrations of our chemical library. We found that all the compounds that delayed zebrafish embryonic development also inhibited cell proliferation, although the rank of potency for some of the compounds differed slightly (data not shown). From both assays, compound 13-1-e remained as the top candidate. We therefore performed additional experiments to elucidate its mechanism of action and ability to inhibit tumor growth *in vivo*. To determine if 13-1-e has affect on cell cycle we isolated primary cells from the control and treated zebrafish embryos and analyzed cell cycle progression by fluorescence activated cell sorting (FACS) analysis. As shown in Figure S1, compound 13-1-e arrested the cell cycle at G1 phase in a dose dependent manner (Figure S1, Table S1).

Several previous studies suggest that embryonic eye development is indicative of cell cycle activity in zebrafish. Inhibition of cyclin D1 protein translation in zebrafish by morpholino knockdown caused more visible defects in embryonic eyes[48]. *In situ* hybridization data showed that expression of CDK2 appears more in embryonic head[49], suggesting that inhibition of CDK2 will affect head structures more than other parts of the embryo. Consistent with this hypothesis, embryos treated with 13-1-e indeed had stronger phenotypes in brain and eyes of zebrafish embryos (Figure 2C).

**Figure 3:**
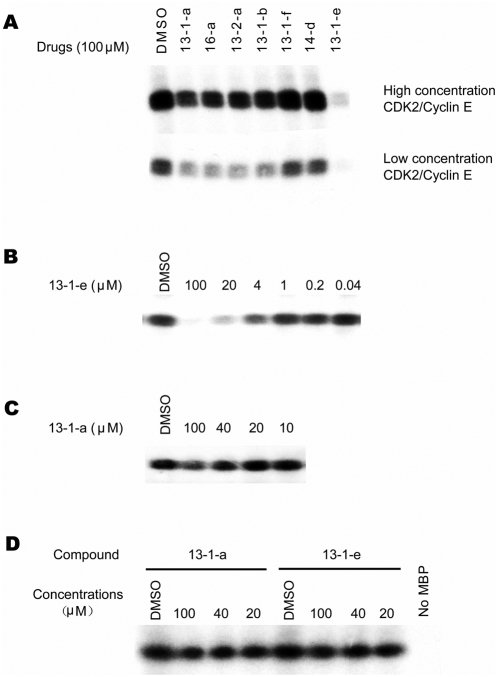
Kinase Assays. (A) The effects of compounds on purified recombinant CDK2/Cyclin E kinase activity to Histone H1; (B) The dose effect of 13-1-e on CDK2/Cycin E kinase activity; (C) The Dose effect of 13-1-a on CDK2/Cyc E kinase activity; (D) The Effect of 13-1-a and 13-1-e on the ERK2 kinase activity towards MBP.

The candidate cell cycle inhibitors were further examined for their ability to inhibit the kinase activity of purified cyclin E/CDK2 using histone H1 as the substrate. Similar to the inhibitory activity on zebrafish embryos and cell proliferation, our study showed that 13-1-e has the highest potency of inhibiting the cyclin E/CDK2 kinase activity, with IC50 between 1–2 µM, whereas compound 13-1-a is moderate (IC50: about 100 µM) and compound 13-1-f has no discernible effect (Figure 3A–C). Analysis by computational docking and simulation indicated that while 13-1-a can form two hydrogen bonds with leucine 83 (L83), 13-1-e forms an additional hydrogen bond with the backbone of glutamic acid 81 (E81) in CDK2. The enhanced inhibition of the kinase activity of cyclin E/CDK2 by 13-1-e is thus likely due to the formation of this additional hydrogen bond between the compound and CDK2. This hypothesis is further confirmed by the observation that 13-1-f has no obvious inhibitory activity to CDK2, which is consistent with our modeling analysis that the replacement of the hydroxyl group by the methoxy group of D ring eliminates this additional hydrogen bond and causes slightly steric interaction with the carbonyl oxygen of E81. To test whether the hydrophobic interaction between the aromatic ring of 13-1-e and Phe80 are critical for the specificity of CDK2 kinase inhibition, we determined the inhibitory specificity of the 13-1-a and 13-1-e towards ERK2, a member of MAP kinase family, also a member of CMGC superfamily, which contains a polar gatekeeper residue glutamine (Q103) at corresponding positions of Phe80 in CDK2. Our analysis indicated that neither 13-1-e nor 13-1-a has significant inhibitory effect on the activity of ERK2 (Figure 3D), although a slight effect was observed at high drug concentrations (>100 µM). Finally, these compounds were profiled against a panel of 21 kinases for inhibitory effect and 13-1-e was shown to have the highest inhibition of CDK2 activity (Table S2).

**Figure 4:**
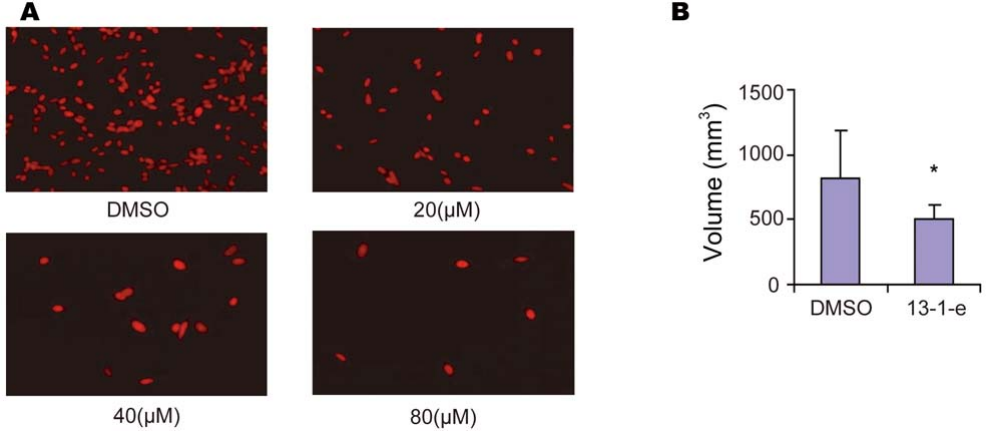
*In vivo* anti-tumor activity. (A) Compound 13-1-e inhibits proliferation of mouse colon tumor cell line CT26 *in vitro*. The number of cells treated with 0.3% DMSO serves as the control here. Red fluorescence represents the nuclei revealed by PI staining. (B) Average tumor size of CT26 cells injected in five mice followed by injections of ten doses of DMSO (50 µl) or 13-1-e (dissolved in 50 µl DMSO, 12.5 mg/kg for every two days) are 821 (±345) mm^3^ (DMSO) and 510 (±107) mm^3^ (13-1-e). P<0.05.

To test if 13-1-e was able to inhibit tumor cell proliferation *in vitro* and tumor size *in vivo*, two more experiments were carried out. First, in cultured condition, 13-1-e was shown to inhibit proliferation of mouse colon tumor cells CT26 with an IG50 of approximately 20 µM (Figure 4A). Second, in a xenograft tumor model, ten days after subcutaneous injection of 3×10^5^ CT26 into the BalB/C mice, 12.5 mg/kg of 13-1-e was intraperitoneally administrated every other day for each mouse (N = 5). As shown in Figure 4B, tumor sizes were significantly smaller than those of controls injected with dimethyl sulfoxide (DMSO) after 10 treatments. Under this condition, mice treated 13-1-e had no obvious detrimental phenotypes. These studies established that 13-1-e has the ability to inhibit cancerous cell growth both *in vitro* and *in vivo*.

## Discussion

Our compound is likely inhibiting CDK as one of its major targets, although other kinases may be involved. It has been shown that CDKs are highly conserved proteins throughout the evolution. In particular, zebrafish and human CDK2 proteins are very similar to each other, with both of them having 298 amino acid residues and sharing about 90% identities of amino acid residues. Figure 1E summarizes the chemical structures of quinoline-based tetracycles used in the inhibition assay of zebrafish embryonic growth. Analysis of the activities of these compounds suggests that several compounds in 13-1 family, such as 13-1-a, 13-1-e are all growth inhibitors. Among them 13-1-e shows the most promising effects on the zebrafish embryonic growth, the selective inhibition of cyclin E/CDK2 kinase activity, and the prevention of tumor cell proliferation in vitro and tumor size in vivo. These observations are in agreement with the computational analysis of our previous docking study, which indicates compound 13-1-e can fit the adenine binding pocket well with three hydrogen bond sites. Other quinoline-based tetracyclic scaffolds of series 13-2 to 17 are mostly ineffective in the zebrafish embryonic assays. It is worth noting that 13-1-a and 13-1-f both are capable of inhibiting embryonic growth but appear not effective for inhibiting CDK2. These compounds may have different targets in vivo that regulate cell proliferation.

Although our lead compound is still less potent and selective compared to some of the previously reported CDK2 inhibitors 13-1-e and its derivatives represent a new scaffold for improvement. This scaffold is a promising template for the rational design and synthesis of novel kinase inhibitors. Considering their low molecular weights, this chemical class will allow facile and broad decoration with various substituents on their framework. With such a high spatial potential of improving this scaffold it is possible to generate more potent and selective CDK inhibitors by exploiting additional interactions with residues that lie outside the ATP binding cleft. As a potential candidate for cancer therapies inhibition of excess cell proliferation without strong toxicity will be the ultimate goal. Our study demonstrates the utility of an integrated approach to rapidly identify novel scaffolds that have activity of inhibiting cell proliferation. With this approach, growth inhibition activity of any newly synthesized compounds can be determined within 24 hours using living zebrafish embryos. In the case of 13-1-e, although CDK2 may not be the only target, it may have better potential to block cancerous cell growth by targeting more than one kinase.

Our studies establish that zebrafish embryo assay can be used to rapidly screen for cell cycle and proliferation inhibitors. This assay, coupled with new chemical synthesis, computational, biochemical and mammalian analysis, allows the identification of specific small molecules that inhibit specific kinase such as CDK2. The compound we identified inhibits zebrafish embryo growth at a specific cell cycle phase, reduces mammalian CDK2 activity as well as tumor cell proliferation *in vitro* and *in vivo*. Since we only select those compounds that retard embryonic growth but not induce gross abnormality of embryonic body and tissues, they may have less toxicity in further preclinical studies. The zebrafish model for identifying kinase inhibitors should have even broader applications. The recent study by Lemeer *et al.* suggests that a large number of protein kinase activities in developing embryos can be profiled using arrays of multiple standard peptide substances, suggesting a wide conservation of kinases between human and zebrafish[50]. With direct analysis of zebrafish embryos treated with a specific small molecule, it should be possible to determine its potency and specificity through phenotypic determination coupled with profiling a panel of representative kinases.

## Materials and Methods

### Synthetic protocols

Reference [44].

### Chemical treatment

Embryo treatments were done from 5.5 hours post fertilization (hpf) until 24 hpf in Holtfreter's buffer with 1% DMSO at 28.5°C. Cell line testing of compounds was performed in medium with 0.3% DMSO at 37°C. Holtfreter's Solution: NaCl 3.5 g, NaHCO_3_ 0.2 g, KCl 0.05 g, MgSO_4_ stock solution 333 µl, CaCl_2_ stock solution 333 µl, add ddH_2_O 1 liter (MgSO_4_ stock solution: MgSO_4_ 300 g in 500 ml ddH_2_O; CaCl_2_ stock solution: CaCl_2_ 150 g in 500 ml ddH_2_O), adjust pH to 7.3.

### Screen methods

The primary screen was carried out in 96-well-plates. 2 µL of each compound (2.5 mg/mL in DMSO) was diluted to a total volume of 200 µL with Holtfreter's buffer. The average concentration of each small molecule was about 100 µM. Embryos are distributed to 96-well-plates with three embryos placed in each well. Then diluted solution of each compound was added. Embryos were exposed to compound solution and incubated at 28.5°C from 5.5 hpf until 24 hpf. Phenotypes were observed for two times at 12 hpf and 24 hpf separately. Once active compounds were identified, each one was analyzed at 100, 40, 30, 20, 10, 4, 2, and 0.4 µM to determine its lowest effective concentration.

### Image acquisition and analysis

For general examination, GFP-positive embryos or larvae were viewed under an Axioimager Z1 fluorescence microscope (Zeiss), equipped with 5, 10 and 20× objectives.

### DNA content analysis

Dechorionated embryos were disaggregated on ice in 1×PBS and serially passed through 105- and 40-µm filters. Cells were washed in ice-cold 1×PBS and stained with propidium iodine. Fluorescence-activated cell sorter (FACS) analysis was done on a BD FACSCalibur flow cytometry (Becton Dickinson, Franklin Lakes, NJ, USA) and analyzed using CellQuest.

### Kinase assays

The CDK2/GST-Cyclin E kinase complex was produced in baculovirus expression system. The purified kinase (100 ng) was mixed with 2 mg histone H1(Upstate), 4 ml of 5× kinase buffer(100 mM Tris, pH 7.4, 50 mM MgCl_2_, 2.5 mM DTT), 0.5 mCi gamma-^32^P-ATP, the indicated amount of chemicals and water to a final volume of 20 ml. The mixtures were incubated at 30°C for 30 min and then loaded to an SDS-PAGE and the labeled histone H1 was separated by electrophoresis and visualized by autoradiography. The HA-tagged ERK2 was expressed in 293 cells after transfection and the kinase was immunoprecipitated with anti-HA antibody/protein A Sepharose beads. The beads were assayed for Erk2 kinase activity in the presence or absence of chemicals as conducted in CDK assays, except 2 mg MBP (Sigma) were used instead of histone H1. Inhibition profile assay against a panel of 21 kinases was carried out by a commercial service with a fluorescence assay using 5 µM of each compound.

### Determination of GI50 in cultured cells

MDA-MB-231 or CT26 cells were plated at a density of 2000 cells/well in a 96-well plate. Drugs were added after 24 h and serial diluted (4, 7.5, 15, 20, 40, 80, 150 µM). The cells were incubated with the compounds at 37°C for 72 h, and then the cell number was measured.

### *In vivo* anti-tumor activity

3×10^5^ CT26 cells were subcutaneously injected into the right flank of 6–8 weeks old BalB/C female mice. When tumors were palpatable at 11 days after inoculation (4–6 mm in diameter), mice were divided into five groups (N = 5 for each group). These mice were given intraperitoneally with 50 µl PBS, 50 µl DMSO, or each chemical compounds dissolved in 50 µl DMSO, respectively. Reagents [12.5 mg/kg; total 50 µl] were given every 2 days for 20 days. Tumor size was determined by caliper measurement of the largest and perpendicular diameters and volumes were calculated according to the formula: tumor volume (mm^3^) = 0.52×length (mm)×width (mm)×width (mm).

## Supporting Information

Figure S1 Cell cycle analysis of embryos treated with 13-1-e 40 µmol/L compound 13-1-e could arrest cell cycle at G1 phase in zebrafish embryos compare with 1%DMSO control. Embryos were treated with compound from 7 hpf, after exposed to compounds for 3 hours, embryos were manipulated to do the FACS analysis.
(1.53 MB TIF)

Table S1 The cell cycle phase changes depend on concentrations of 13-1-e Embryos were treated with deferent concentrations of 13-1-e since 7 hpf; after exposed to compounds for 3 hours, embryos were manipulated to do the FACS analysis. (Based on three independent experiments)
(0.03 MB DOC)

Table S2 Inhibition of individual kinases by three selected compounds Each compound (5 µM) was added to individual kinase and activity was compared to control activity without compounds. Numbers indicate % of inhibition. Note that 13-1-e inhibited CDK2 by 45%, which is the highest among the 21 kinases. Interestingly, the compounds appear to stimulate activities of some kinases.
(0.05 MB DOC)
