# Supplementary figures and images for: Identifying Tumor Cell Growth Inhibitors by Combinatorial Chemistry and Zebrafish Assays
Source: PLoS One. 2009 Feb 5;4(2):e4361. doi: 10.1371/journal.pone.0004361 (PMC2633036; doi:10.1371/journal.pone.0004361)

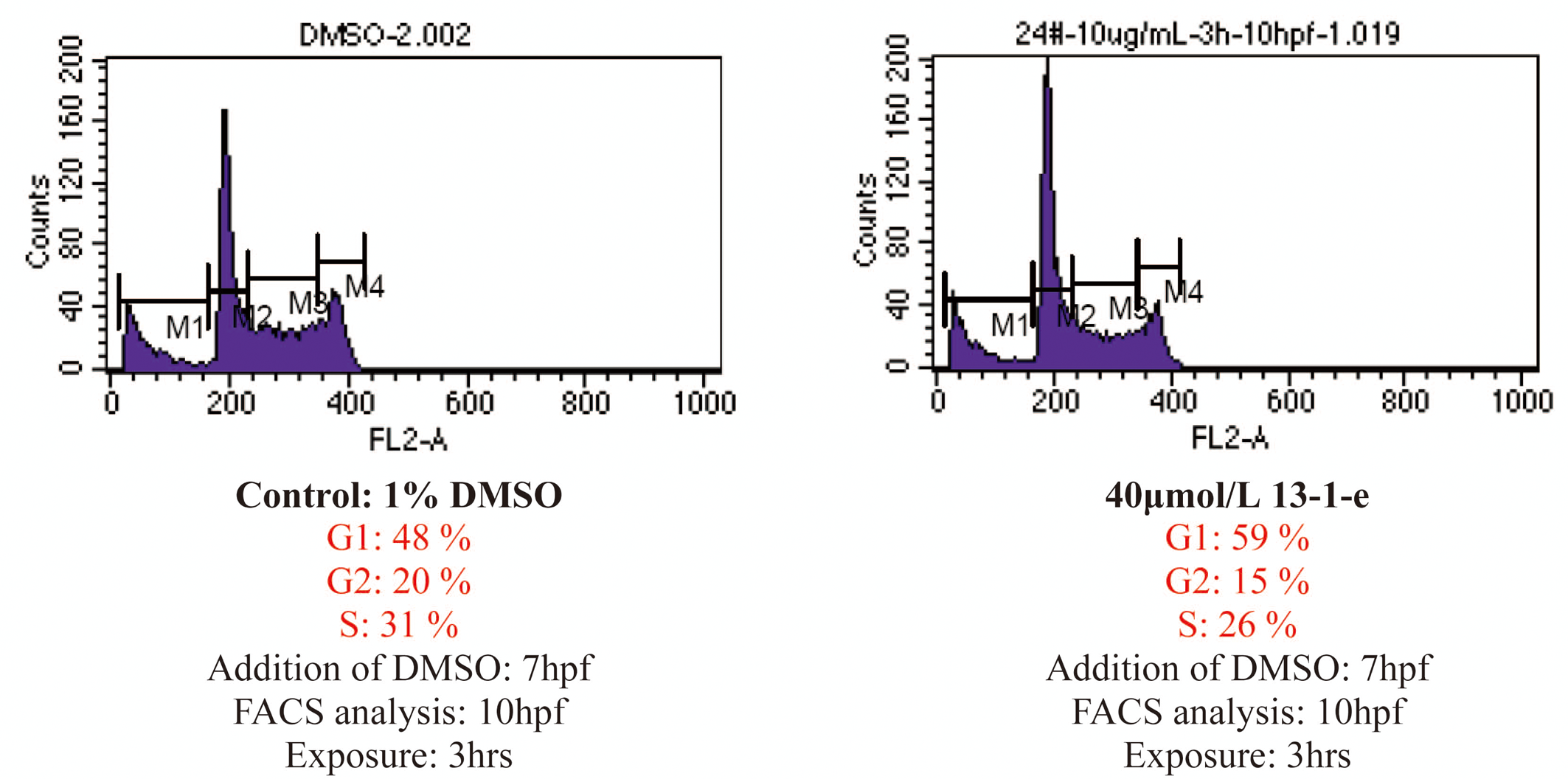

Supplement: Figure S1 — Cell cycle analysis of embryos treated with 13-1-e 40 µmol/L compound 13-1-e could arrest cell cycle at G1 phase in zebrafish embryos compare with 1%DMSO control. Embryos were treated with compound from 7 hpf, after exposed to compounds for 3 hours, embryos were manipulated to do the FACS analysis. (1.53 MB TIF) [file pone.0004361.s001.tif]
