# Supplementary material for: Identifying Tumor Cell Growth Inhibitors by Combinatorial Chemistry and Zebrafish Assays
Source: PLoS One. 2009 Feb 5;4(2):e4361. doi: 10.1371/journal.pone.0004361 (PMC2633036; doi:10.1371/journal.pone.0004361)
Supplement: Table S1 — The cell cycle phase changes depend on concentrations of 13-1-e Embryos were treated with deferent concentrations of 13-1-e since 7 hpf; after exposed to compounds for 3 hours, embryos were manipulated to do the FACS analysis. (Based on three independent experiments) (0.03 MB DOC) [file pone.0004361.s002.doc]

### Table S1 –The cell cycle phase changes depend on concentrations of 13-1-e

|  | G1 | S | G2/M |
| --- | --- | --- | --- |
| 1% DMSO | 48 (±2)% | 31 (±1)% | 20 (±1)% |
| 13-1-e) 20 μM | 53 (±1)% | 24 (±2)% | 23 (±3)% |
| 13-1-e) 30 μM | 55 (±1)% | 28 (±2)% | 17 (±1)% |
| 13-1-e) 40 μM | 59 (±2)% | 26 (±1)% | 15 (±1)% |

Embryos were treated with deferent concentrations of 13-1-e since 7 hpf; after exposed to compounds for 3 hours, embryos were manipulated to do the FACS analysis. (Based on three independent experiments)
