# Supplementary material for: Identifying Tumor Cell Growth Inhibitors by Combinatorial Chemistry and Zebrafish Assays
Source: PLoS One. 2009 Feb 5;4(2):e4361. doi: 10.1371/journal.pone.0004361 (PMC2633036; doi:10.1371/journal.pone.0004361)
Supplement: Table S2 — Inhibition of individual kinases by three selected compounds Each compound (5 µM) was added to individual kinase and activity was compared to control activity without compounds. Numbers indicate % of inhibition. Note that 13-1-e inhibited CDK2 by 45%, which is the highest among the 21 kinases. Interestingly, the compounds appear to stimulate activities of some kinases. (0.05 MB DOC) [file pone.0004361.s003.doc]

### Table S2 –Inhibition of individual kinases by three selected compounds

|  | 13-1-a | 13-1-e | 13-1-f |
| --- | --- | --- | --- |
| ABL1 | 28 | -6 | 8 |
| CAMK1D (CaMKI delta) | 9 | -5 | -4 |
| CAMK2B (CaMKII beta) | -67 | -7 | -23 |
| CDK2/cyclin A | 7 | 45 | -2 |
| CHEK2 (CHK2) | -6 | -29 | -22 |
| CLK1 | 5 | 17 | -2 |
| CSNK1D (CK1 delta) | 9 | 4 | -34 |
| FLT3 | 18 | 30 | 18 |
| KDR (VEGFR2) | 37 | 35 | 34 |
| LCK | 4 | 4 | 3 |
| MAP2K1 (MEK1) | 7 | 10 | 7 |
| MAP3K9 (MLK1) | 9 | -29 | 5 |
| MAPK1 (ERK2) | 3 | -7 | -2 |
| MAPK8 (JNK1) | 1 | 6 | 4 |
| MAPKAPK2 | 11 | 0 | -4 |
| MYLK2 (skMLCK) | 12 | 0 | -1 |
| NEK2 | 8 | -10 | 2 |
| PLK1 | 1 | 6 | 1 |
| RPS6KA1 (RSK1) | 6 | 13 | -18 |
| SGK (SGK1) | 43 | 14 | 20 |
| STK3 (MST2) | 1 | -2 | 3 |

Each compound (5 μM) was added to individual kinase and activity was compared to control activity without compounds. Numbers indicate % of inhibition. Note that 13-1-e inhibited CDK2 by 45%, which is the highest among the 21 kinases. Interestingly, the compounds appear to stimulate activities of some kinases.
